# Surgical Treatment of Spheno-Orbital Meningiomas: A Systematic Review and Meta-Analysis of Surgical Techniques and Outcomes

**DOI:** 10.3390/jcm12185840

**Published:** 2023-09-08

**Authors:** Edoardo Agosti, Marco Zeppieri, Lucio De Maria, Marcello Mangili, Alessandro Rapisarda, Tamara Ius, Leopoldo Spadea, Carlo Salati, Alessandro Tel, Antonio Pontoriero, Stefano Pergolizzi, Filippo Flavio Angileri, Marco Maria Fontanella, Pier Paolo Panciani

**Affiliations:** 1 Division of Neurosurgery, Department of Surgical Specialties, Radiological Sciences and Public Health, University of Brescia, 25123 Brescia, Italy; edoardo_agosti@libero.it (E.A.);; 2 Department of Ophthalmology, University Hospital of Udine, Piazzale S. Maria Della Misericordia 15, 33100 Udine, Italy; 3 Department of Neurosurgery, Fondazione Policlinico Universitario Agostino Gemelli IRCSS, 00168 Rome, Italy; 4 Neurosurgery Unit, Head-Neck and NeuroScience Department, University Hospital of Udine, p.le S. Maria Della Misericordia 15, 33100 Udine, Italy; 5 Eye Clinic, Policlinico Umberto I, “Sapienza” University of Rome, 00142 Rome, Italy; 6 Clinic of Maxillofacial Surgery, Head-Neck and NeuroScience Department University Hospital of Udine, p.le S. Maria Della Misericordia 15, 33100 Udine, Italy; 7 Radiation Oncology Unit, Department of Biomedical, Dental Science and Morphological and Functional Images, University of Messina, 98125 Messina, Italy; 8 Neurosurgery Unit, Department of Biomedical, Dental Science and Morphological and Functional Images, 98125 Messina, Italy

**Keywords:** spheno-orbital meningiomas, systematic review, meta-analysis, surgical approaches, clinical outcomes, surgical outcomes

## Abstract

Background: Spheno-orbital meningiomas (SOMs) are rare tumors arising from the meninges surrounding the sphenoid bone and orbital structures. Surgical resection is the primary treatment approach for SOMs. Several surgical approaches have been described during the decades, including microsurgical transcranial (MTAs), endoscopic endonasal (EEAs), endoscopic transorbital (ETOAs), and combined approaches, and the choice of surgical approach remains a topic of debate. Purpose: This systematic review and meta-analysis aim to compare the clinical and surgical outcomes of different surgical approaches used for the treatment of SOMs, discussing surgical techniques, outcomes, and factors influencing surgical decision making. Methods: A comprehensive literature review of the databases PubMed, Ovid MEDLINE, and Ovid EMBASE was conducted for articles published on the role of surgery for the treatment of SOMs until 2023. The systematic review was performed according to the Preferred Reporting Items for Systematic Reviews and Meta-Analysis guidelines. Meta-analysis was performed to estimate pooled event rates and assess heterogeneity. Fixed- and random-effects were used to assess 95% confidential intervals (CIs) of presenting symptoms, outcomes, and complications. Results: A total of 59 studies comprising 1903 patients were included in the systematic review and meta-analysis. Gross total resection (GTR) rates ranged from 23.5% for ETOAs to 59.8% for MTAs. Overall recurrence rate after surgery was 20.7%. Progression-free survival (PFS) rates at 5 and 10 years were 75.5% and 49.1%, respectively. Visual acuity and proptosis improvement rates were 57.5% and 79.3%, respectively. Postoperative cranial nerve (CN) focal deficits were observed in 20.6% of cases. The overall cerebro-spinal fluid (CSF) leak rate was 3.9%, and other complications occurred in 13.9% of cases. MTAs showed the highest GTR rates (59.8%, 95%CI = 49.5–70.2%; *p* = 0.001) but were associated with increased CN deficits (21.0%, 95%CI = 14.5–27.6%). ETOAs had the lowest GTR rates (23.5%, 95%CI = 0.0–52.5%; *p* = 0.001), while combined ETOA and EEA had the highest CSF leak rates (20.3%, 95%CI = 0.0–46.7%; *p* = 0.551). ETOAs were associated with better proptosis improvement (79.4%, 95%CI = 57.3–100%; *p* = 0.002), while anatomical class I lesions were associated with better visual acuity (71.5%, 95%CI = 63.7–79.4; *p* = 0.003) and proptosis (60.1%, 95%CI = 38.0–82.2; *p* = 0.001) recovery. No significant differences were found in PFS rates between surgical approaches. Conclusion: Surgical treatment of SOMs aims to preserve visual function and improve proptosis. Different surgical approaches offer varying rates of GTR, complications, and functional outcomes. A multidisciplinary approach involving a skull base team is crucial for optimizing patient outcomes.

## 1. Introduction

**Figure 1:**
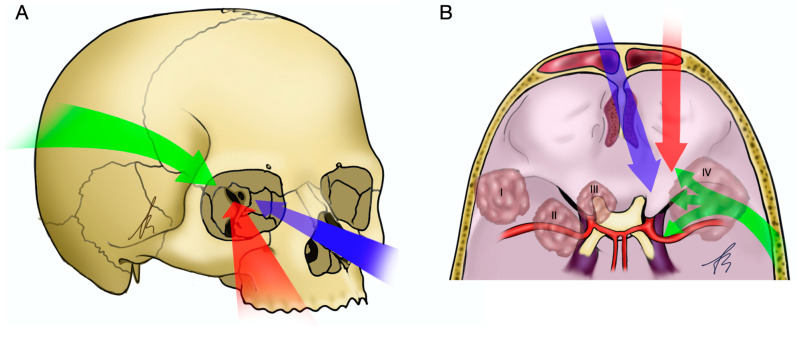
Graphical illustration of the surgical corridors of MTAs, EEAs, and ETOAs and anatomical classes of SOMs. (**A**) Anterolateral view of a skull: MTAs (green arrow), EEAs (blue arrow), and ETOAs (red arrow). (**B**) Supero-posterolateral view of the skull base. MTAs can provide several surgical corridors to different portion of the spheno-orbital region, including cavernous sinus, SOF and orbital apex, and anterior cranial fossa. SOMs anatomical classes are also here represented: anatomical class I (lateral or superolateral SOMs), II (medial or inferomedial SOMs), III (orbital apex SOMs), and IV (diffuse SOMs).

Spheno-orbital meningiomas (SOMs) are rare tumors, accounting for 0.2% and 9% of all meningiomas, arising from the meninges surrounding the sphenoid bone and orbital structures [1,2]. These tumors pose significant challenges due to their anatomical location and proximity to critical structures, necessitating a multidisciplinary approach to management [3]. Over the years, various surgical approaches have been developed and utilized, including microsurgical transcranial (MTAs), endoscopic endonasal (EEAs), endoscopic transorbital (ETOAs), and combined approaches (Figure 1). Each approach has its unique advantages and limitations, and there is a need to comprehensively compare their clinical and surgical outcomes to guide treatment decisions [4,5].

Surgical resection is the primary goal in the treatment of SOMs, aiming for gross total resection (GTR) to achieve optimal oncological control. However, the choice of surgical approach can have a significant impact on the extent of resection and postoperative outcomes. Additionally, postoperative complications and progression-free survival (PFS) are important outcome measures to assess the overall success of the surgical intervention [3,6].

The objective of this systematic literature review and meta-analysis is to compare the clinical and surgical outcomes among patients undergoing MTAs, EEAs, ETOAs, and combined approaches for the surgical treatment of SOMs. By examining the existing evidence, this study aims to provide clinicians with valuable insights into the advantages and limitations of each approach, and facilitate evidence-based decision making in the management of these challenging tumors.

## 2. Materials and Methods

### 2.1. Literature Search

The systematic review was performed according to the Preferred Reporting Items for Systematic Reviews and Meta-Analysis (PRISMA) guidelines [7]. A comprehensive literature search of the databases PubMed, Ovid MEDLINE, and Ovid EMBASE was designed and conducted by an experienced librarian with input from the authors. The keywords “spheno-orbital”, “meningioma”, and “approach”, were used in “AND” and “OR” combinations. The following research string was used: “((spheno-orbital or sphenoorbital) AND (meningioma) AND (approach OR surgery OR microsurgical OR endoscopic OR endonasal OR transorbital OR combined) AND (outcome OR resection OR survival OR complication OR deficit))”. The last search for articles pertinent to the topic was conducted on 1 July 2023. Other pertinent articles were retrieved through reference analysis. Two authors (E.A. and L.D.M.) independently conducted the abstract screening for eligibility. Any discordance was solved by consensus with two senior authors (M.Z. and P.P.P.). No restrictions on the date of publication were made. Exclusion criteria were as follows: studies published in languages other than English, preclinical anatomical and laboratory studies, studies which include patients with SOMs not surgically treated, meta-analysis, and literature review. Inclusion criteria: studies reporting at least a case of SOM surgically treated. The study was not registered, thus, there is no registration number.

### 2.2. Data Extraction

For each study, we abstracted the following baseline information: author, country, journal, title, and year of publication; design and period in which the population was collected; sample size, mean and range of age, percentage of female; histology and grade of the lesion (according to WHO classification 2021); clinical presentations, including visual acuity decrease, proptosis, cranial nerves (CNs) deficits, and other signs and symptoms; number and percentages of patient who received gross total resections, adjuvant radiotherapy (RT), other adjuvant therapies; follow-up period.

### 2.3. Outcomes

Outcomes were meta-analyzed based on the type of surgical approach (MTA, EEA, ETOA, or combined). The outcomes were also tested to evaluate any statistically significant differences according to the anatomical site and extension of the SOM and according to the WHO grade (grade I, II, and III, according to WHO classification 2021). Based on site, SOMs were divided into four categories, specifically, superior or superolateral, inferior or inferomedial, apex, and diffuse.

Our primary outcomes were GTR, progression-free survival at 5 years (PFS 5-y) and at 10 years (PFS 10-y), and recurrences rate. Secondary outcomes were improvement of visual acuity, improvement of proptosis, postoperative CNs deficits, postoperative cerebrospinal fluid (CSF) leak, and other complications.

### 2.4. Study Risk of Bias Assessment

**Figure 2:**
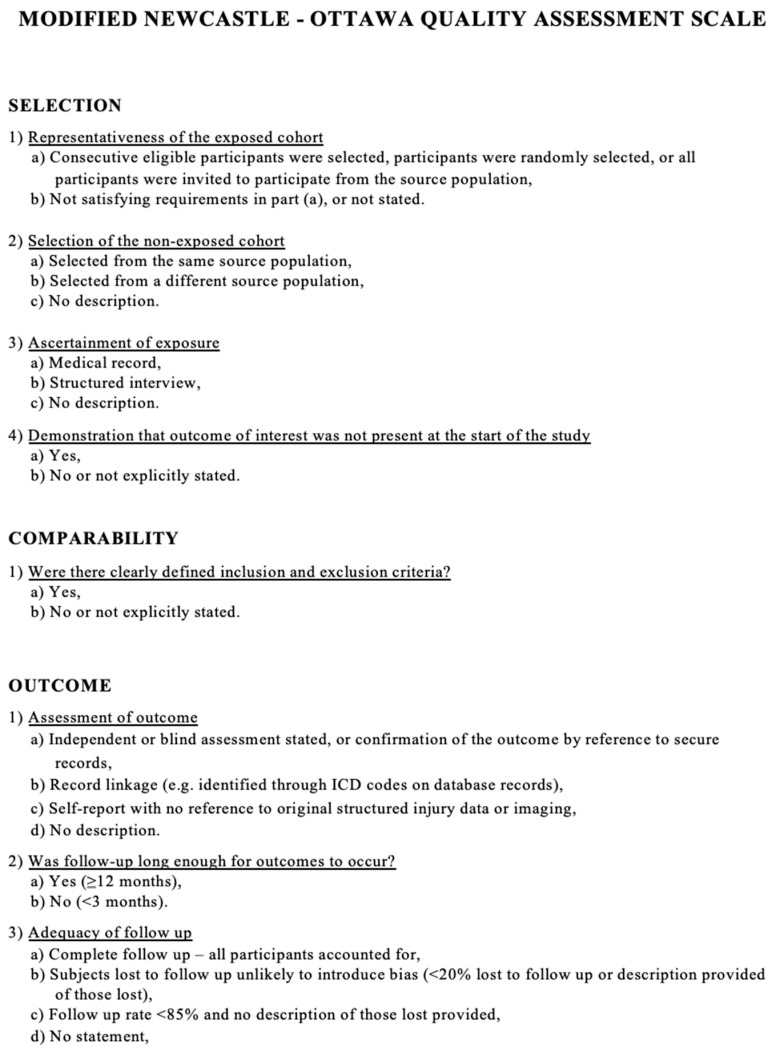
Modified Newcastle–Ottawa scale used to assess the methodologic quality of the studies included in our meta-analysis.

We modified the Newcastle–Ottawa scale (NOS) to assess the methodologic quality of the studies included in our meta-analysis. This tool is designed for use in comparative studies. However, as there was no control group in our studies, we assessed their methodologic quality based on selected items from the scale, focusing on the following questions: (1) Did the study include all patients or consecutive patients vs. a selected sample? (2) Was the study retrospective or prospective? (3) Was clinical follow-up satisfactory, thus allowing ascertainment of all outcomes? (4) Were outcomes reported? (5) Were there clearly defined inclusion and exclusion criteria? (Figure 2).

### 2.5. Statistical Analysis

Descriptive statistics were reported, including ranges and percentages. For the purpose of the meta-analysis, we estimated from each cohort the cumulative prevalence and 95% confidence interval for each outcome. Event rates were pooled across studies with a random-effects meta-analysis. Heterogeneity across studies was evaluated using the I2 statistic. An I2 value of >50% suggests substantial heterogeneity. For formal statistical comparisons and subgroup analysis, we also extracted a chi-square contingency table to calculate *p* values. The level of statistical significance was set to *p* < 0.05. Meta-regression was not used in this study. Statistical analyses were performed using OpenMeta Analyst http://www.cebm.brown.edu/openmeta accessed on 20 June 2023) and the R statistical package v3.4.1 http://www.r-project.org (accessed on 20 June 2023).

## 3. Results

### 3.1. Literature Review

**Figure 3:**
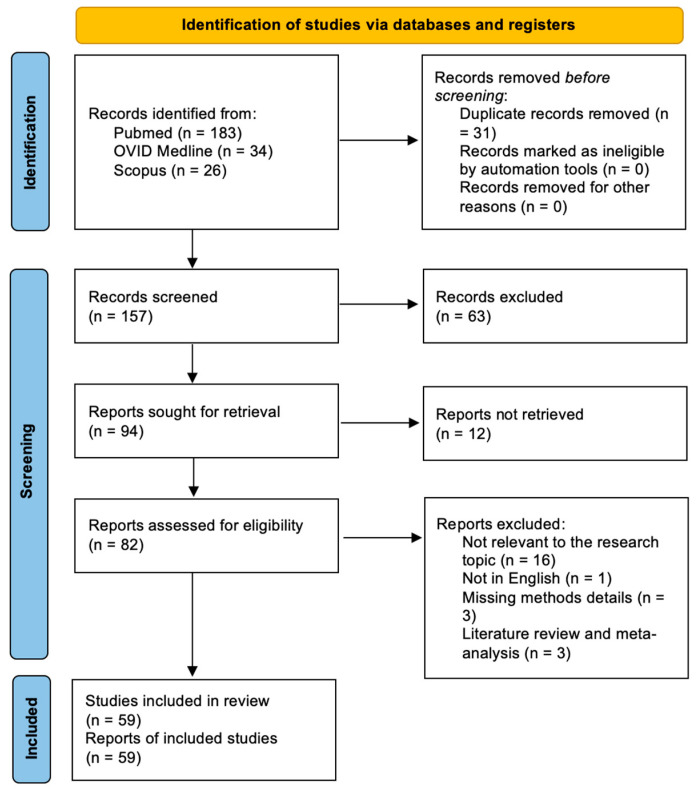
PRISMA flow diagram depicting the literature search process.

A total of 157 papers were identified after duplicate removal. After title and abstract analysis, 94 articles were identified for full-text analysis. Eligibility was ascertained for 82 articles. The remaining 23 articles were excluded for the following reasons: (1) not relevant to the research topic (16 articles), (2) not in English (1 article), lack of method details (3 articles), systematic literature review or meta-analysis (3 articles). All studies included in the analysis had at least one or more outcome measures available for one or more of the patient groups analyzed. Figure 3 shows the flow chart according to the PRISMA statement.

### 3.2. Baseline Data

A summary of the included studies is provided in Table 1. All studies included in our systematic review were retrospective. The study periods ranged from 1958 to 2021. A total of 1903 patients were included. The mean age at surgery ranged from 34 to 62 years. The WHO grade was reported in 31 studies (52%). At presentation, 1385/1730 patients had proptosis (80%), 920/1773 patients (52%) had a visual acuity decrease, and 191/1156 had CN deficits (13%). Regarding treatment, 875/1542 underwent GTR (57%) and 291/1420 received post-op RT (41%). The mean follow-up time ranged from 2 to 135 months.

### 3.3. Efficacy Outcomes

**Figure 4:**
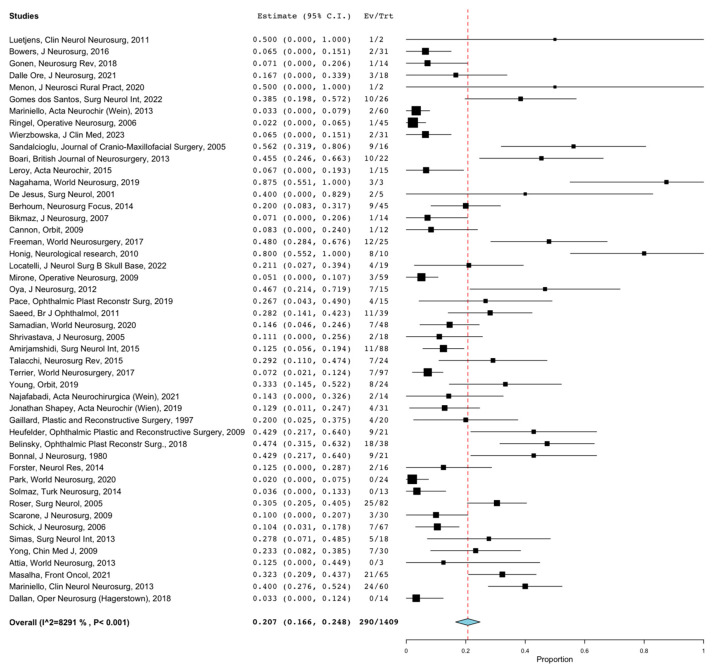
Forest plot of overall recurrence rates. (CIs = confidential intervals).

**Figure 5:**
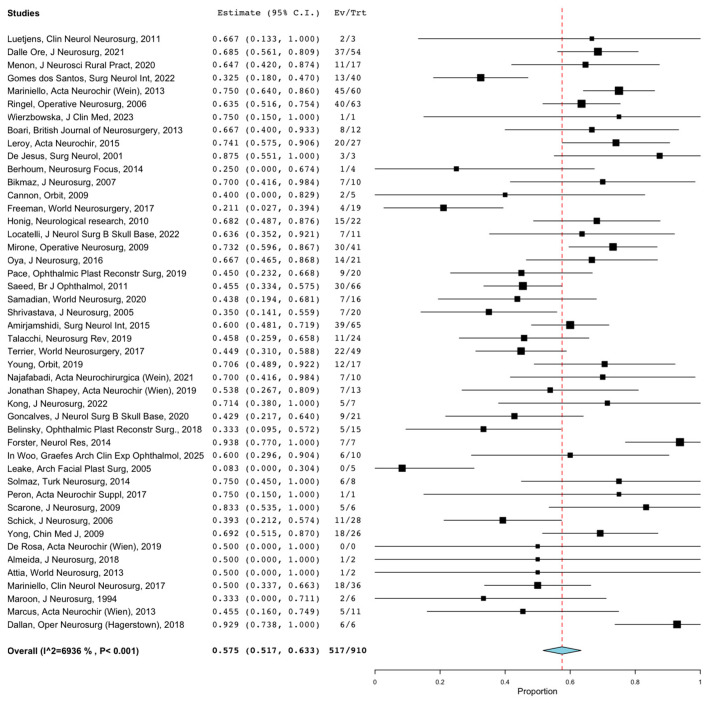
Forest plot of overall visual acuity improvement rates. (CIs = confidential intervals).

Overall GTR rates were reported in 1542 patients. The overall rate of GTR following SOMs resection through any surgical approach was 57.3% (95%CI = 47.5–67.1%). Lesions treated through the MTA and anatomical class I lesions had the highest GTR rate at 59.8% (95%CI = 49.5–70.2%; *p* = 0.001) and 78.6% (95%CI = 60.1–97.1%; *p* = 0.001), while lesions treated through ETOA combined with EEA and WHO grade I lesions had the lowest GTR rate at 23.5% (95%CI = 0–52.5%; *p* = 0.001) and 43.1% (95%CI = 20.4–65.9%; *p* = 0.001). Overall recurrence rates were reported in 1409 patients. The overall rate of recurrence following SOMs resection through any surgical approach was 20.7% (95%CI = 16.6–24.8%). Figure 4 shows the forest plot of overall recurrence rates. Recurrence rates ranged from 4.4% (95%CI = 0–11.2%) for lesions treated through ETOA to 24.4% (95%CI = 19.4–29.4%) for lesions treated through MTA (*p* = 0.014). The overall rates of PFS 5-y and PFS 10-y were reported in 230 and 159 patients, and were 75.5% (95%CI = 70–81.1%) and 49.1% (95%CI = 41.3–56.8%), respectively. The overall rates of visual acuity and proptosis improvement were reported in 910 and 1132 patients and were 57.5% (95%CI = 51.7–63.3%) and 79.3% (95%CI = 73.7–84.8%), respectively. Figure 5 shows the forest plot of overall visual acuity improvement rates. Anatomical class I lesions had the highest visual acuity improvement rate at 71.5% (95%CI = 63.7–79.4%; *p* = 0.003). Lesions treated through the ETOA and anatomical class I lesions had the highest proptosis improvement rates at (60.1%, 95%CI = 38.0–82.2; *p* = 0.001) and 79.4% (95%CI = 57.3–100.0%; *p* = 0.002), respectively.

### 3.4. Safety Outcomes

Overall CN focal deficits and CSF leak rates were reported in 763 and 517 patients, respectively. The overall rate of CN focal deficits was 20.6% (95%CI = 14.9–26.3%). The lowest rate was reported for lesions treated through the ETOA (7.3%; 95%CI = 0–18.1%) and the highest rate was reported for lesions treated through the MTA (21.0%, 95%CI = 14.5–27.6%). The overall rate of CSF leak was 3.9% (95%CI = 2.3–5.5%). The CSF leak rate was highest for lesions treated through the combined ETOA and EEA (20.3%; 95%CI = 0–46.7%; *p* = 0.551) and was the lowest for lesions treated through the MTA (4.9%, 95%CI = 2.8–6.9%). Other complication rates were reported in 1181 patients. The overall rate was 13.9% (95%CI = 10.1–17.7%). The rate of other complications was the lowest for WHO grade I and II lesions (11.7%; 95%CI = 6.5–16.8%; *p* = 0.001). The efficacy and safety outcomes are summarized in Table 2 and Table 3.

### 3.5. Study Heterogeneity

The I2 values were >50%, indicating substantial heterogeneity for the following outcomes: GTR, recurrence, visual acuity improvement, proptosis improvement, CN focal deficits, and other complications. The I2 values were <50%, indicating a lack of substantial heterogeneity for the following outcomes: PFS 5-y, PFS 10-y, and CSF leak.

## 4. Discussion

As far as we know, this is the largest systematic literature review and meta-analysis available in the literature. Clinical and surgical outcomes of SOMs surgically treated have been analyzed. According to our findings, SOMs treated through the MTAs and anatomical class I lesions had the highest GTR rate, while ETOAs either as single or combined approach with EEAs offered the lowest GTR rate. On the other hand, MTAs presented the higher recurrence rates, and no statistically significant differences were detected between the different approaches regarding the PFS 5-y and PFS 10-y. Anatomical class I SOMs and SOMs treated with ETOA showed better rates of postoperative vision acuity and proptosis improvement. MTAs are more prone to postoperative CNs deficits, while combined ETOA and EEA have the highest rate of postoperative CSF leaks.

MTAs are commonly utilized for the surgical treatment of SOMs, with the pterional approach being the most frequently employed [60]. MTAs offer advantages such as wide exposure and the ability to achieve radical resection of hyperostotic bone. Recently, various EEAs and ETOAs, either as stand-alone options or in combination, have been described for SOMs removal [37,44,46,52,59]. EEAs are particularly effective for decompressing the medial part of the optic canal, while ETOAs enable further decompression of the hyperostotic bone and tumor removal, especially in lesions located more laterally [37]. Endoscopic approaches offer less invasive corridors and aesthetically pleasing results. However, due to the limitations in achieving GTR, these approaches should be reserved for selected patients with suspected benign SOMs exhibiting minimal intradural growth [14,60,61]. In such cases, the primary goal is symptom relief through decompression of the optic canal, with subsequent consideration of adjuvant radiotherapy (RT) for any residual tumor.

SOMs manifest as the expansion of the sphenoid bone, extending into the orbit and causing hyperostosis [42]. These tumors often spread to various adjacent areas, such as the sphenoid, orbital roof, middle fossa, superior orbital fissure (SOF), optic canal (OC), anterior clinoid, or cavernous sinus (CS). They can also invade the temporalis or lateral pterygoid muscles [62]. Due to their invasive nature, SOMs exhibit radiologic characteristics resembling malignancies [23]. However, in practical terms, most SOMs are classified as WHO-I tumors. The complete removal of SOMs through surgery is frequently limited by their infiltration into the SOF, CS, extraocular muscles, or cranial nerves [39]. The feasibility of performing aggressive resection has been a subject of debate. Reported rates of GTR in our series was 57.3% (95%CI = 47.5–67.1%). Simpson grade I resection with minimal morbidity is the main treatment goal. However, this often results in significant morbidity to the patient [4,56,63]. For this reason, over time the treatment paradigm has shifted from GTR to aggressive STR as respectful as possible of the healthy neurovascular structures surrounding the lesion [4]. Nowadays, the goal of surgery is, in fact, a symptomatic improvement compared to a GTR, for example, in the case of involvement of the optic canal with the aim of decompressing the optic nerve in order to maximize visual acuity outcomes. Accordingly, limited attempt at resection of meningioma within the cavernous sinus or with SOF involvement is performed given the risk of postoperative CN deficits [4]. This agrees with the data emerging from our study, which showed that anatomical class I lesions had the highest GTR rate, as the cavernous sinus, the orbital apex, and the intraorbital structures were not directly invaded [52,58]. Other examples of surgery aimed at improving the clinical outcome and respectful of the surrounding anatomical structures are reported in the literature. For example, Scarone et al. [20] published a series of 39 patients in which they excluded Simpson I resection in case of SOMs with SOF invasion. Ringel et al. [17] and Boari et al. [28], in a series of 63 and 40 patients, respectively, underline how the intraorbital and SOF extension prevents a GTR, as in the postoperative period there would be a considerable degree of morbidity such as not to justify the complete macroscopic removal of the lesion. Finally, Saeed et al. [29] have sanctified the concept of “symptom-oriented” resection rather than attempted GTR in a personal series of 66 patients [4,17].

According to the literature, proptosis is the most frequently observed preoperative finding and indication for surgery, with a reported occurrence rate of 45–100%. Postoperatively, proptosis improvement has been documented in 52–100% of patients [4,6,14,17,21,24,25,28,29,34,43,64]. Our study aligns with these findings, as we observed an overall clinical presentation of proptosis in 80% of cases. Additionally, the second most commonly reported preoperative finding in the literature is deteriorating visual function, which has been documented in 30–78% of cases [4,6,14,17,21,24,25,28,29,34,43,64]. Our study yielded similar results, with deteriorating visual function observed in 52% of cases. Postoperatively, visual function improvement has been reported in 21–87% of patients [4,6,14,17,21,24,25,28,29,33,34,43,64], consistent with our study’s finding of 57.5% (95%CI = 51.7–63.3%). Furthermore, our study found that 79.3% (95%CI = 73.7–84.8%) of cases exhibited the specified characteristic. Ocular paresis is often the third most common presenting symptom associated with SOMs, in agreement with the data emerging from this review (13%) [3].

The patient’s prognosis and quality of life heavily depend on visual acuity, rendering it a crucial clinical outcome for SOM patients [4]. To improve visual acuity, it is vital to optimize surgical interventions and postoperative follow-up [39]. According to this study, operating on patients, even those with minimal visual impairment or hyperostosis, appears to be beneficial in preventing the development of visual deficits [14,22,32,33,42,65]. The follow-up findings suggest that early surgery is predictive of favorable visual outcomes. Since SOMs tend to invade the bones near the cranial nerve foramina, early surgical intervention may help prevent extensive hyperostosis, narrowing of the foramina, and subsequent cranial nerve deficits [42]. Notably, involvement of the optic canal and intraorbital region has been identified as predictors of postoperative visual deficits. However, it should be noted that surgery itself carries the risk of new visual and cranial nerve deficits. In cases of very elderly patients, individuals with severe comorbidities, or those with extensive disease leading to complete blindness, the potential benefits of surgery may not always outweigh the risks of complications [14]. Nevertheless, in general, the risk of new complications is believed to be lower when patients undergo surgery early in their disease progression, as cranial nerves are less vulnerable when the degree of compression is less severe [54].

Complications following surgery for SOMs commonly include deficits in extraocular movements and trigeminal hypoesthesia [42]. Previous studies have indicated that postoperative deficits in extraocular movements involving CNs III, IV, and VI occur in approximately 7% to 68% of cases [17,25,42]. These findings are generally consistent with the results of this study, which reported a rate of 20.6% (95%CI = 14.9–26.3%). However, the latter figure is closer to the lower end of the range reported in existing literature. While cranial nerve palsies are often temporary, there are cases where they can be permanent. Diplopia, or double vision, tends to be more prevalent among patients who undergo resection of the periorbita [14]. Additionally, trigeminal hypoesthesia is a common comp li ca tion following surgery. Nevertheless, over the years, there has been a decrease in postoperative deficits affecting cranial nerves, likely attributable to a less aggressive surgical approach [3,4].

Over the past three decades, the surgical management of SOMs has undergone signif icant evolution, resulting in improved outcomes and reduced morbidity for patients. In the early 1990s, surgical approaches often involved extensive craniotomies and aggressive tumor resections, aiming to achieve complete tumor removal [66]. While this approach occasionally yielded favorable results, it was associated with considerable risks, such as visual impairment and injury to critical structures. As technological advancements and surgical expertise progressed, the trend shifted towards more conservative strategies in the late 1990s and early 2000s [17]. These techniques, including image-guided surgery and the use of endoscopes, prioritized functional preservation, especially vision, and resulted in reduced complications. By the 2010s, minimally invasive procedures, such as endoscopic endonasal surgery, gained prominence, offering excellent tumor control with minimal morbidity [56]. In 2023, a trend persists in favor of these less invasive techniques, showcasing their efficacy in achieving tumor control while preserving patient quality of life, particularly in terms of visual outcomes [59]. This gradual shift in surgical paradigms highlights the importance of not only eradicating the tumor but also ensuring the best possible functional outcomes for patients with spheno-orbital meningiomas.

Both recent studies and those conducted over 20 years ago provide evidence supporting the utilization of RT for subtotally resected meningiomas, demonstrating improved overall survival and PFS compared to surgery alone [9,17,20,28,64,67,68,69,70]. In cases of disease recurrence and residual tumor progression after primary microsurgery, secondary stereotactic radiosurgery (SRS) is frequently recommended [71]. SRS alone or in combination with hypo-fractionated radiotherapy offers particular advantages for treating SOMs located near the cavernous sinus and orbital apex, where surgical resection is limited, and preserving the neurovascular anatomy around the tumor is of utmost importance [9,17,20,28,64,67,68,69,70]. However, in situations where residual or recurrent lesions are in close proximity to CNs, a single dose of SRS may not be feasible [69]. Consequently, the systematic review highlights that fractionated SRS can serve as an effective approach, ensuring both appropriate aggressiveness towards the residual lesion and protection of the sur rounding neurovascular anatomy. This fractionated SRS approach achieves secondary tumor control while maintaining an acceptable adverse effect profile [71]. Nonetheless, advancements in dose reduction and treatment conformity strategies hold the potential to enhance the feasibility of this option in the future. Furthermore, other radiation modalities, such as external beam radiotherapy (EBRT), intensity-modulated radiotherapy (IMRT), and proton beam radiation therapy (PBRT), are being explored for their early applications in treating SOMs. These alternative radiation techniques offer additional options and potential benefits in the management of SOMs [50,72,73,74]. The role of RT in the treatment of SOMs remains a subject of ongoing debate. This systematic review highlights the lack of a standardized protocol among the authors regarding the use of RT for managing SOMs. According to the findings of this review, it is evident that residual WHO-I tumors do not typically receive secondary RT, regardless of the Simpson grade. However, in cases of recurrent WHO-I tumors, a combination of repeat surgery and postoperative radiotherapy appears to be the most commonly utilized and effective approach for disease control. Adjuvant RT is considered mandatory for WHO-II or WHO-III tumors.^75^ Preliminary evidence suggests that RT may contribute to prolonged PFS, but the decision to administer RT should be carefully evaluated, considering factors such as age, tumor size, and pathology of the residual tumor [14,20].

### Limitations

There are several limitations to the study. This meta-analysis was based primarily on single-center case series and, thus, has limitations inherent to single-center retrospective studies. While we were able to perform subgroup, analyses based on the surgical approach used, we were unable to perform more granular analyses stratifying outcomes by each WHO grade and anatomical class. Nonetheless, our study provides helpful information for providers who are considering surgery for the treatment of SOMs and provides guidance for future areas of investigation.

The limitations of this review stem from a dearth of high-quality studies and significant heterogeneity among those included, which may have constrained our ability to derive definitive conclusions. Moreover, we cannot disregard the possibility of publication bias, as studies reporting positive outcomes or statistically significant results tend to be more readily published. Such bias may have influenced the overall summary effect estimate, potentially leading to an overestimation of the treatment effect. Furthermore, our search strategy may have introduced limitations despite our comprehensive efforts; it is conceivable that some pertinent studies were inadvertently overlooked. Language restrictions and the exclusion of unpublished research may have also contributed to potential bias. Lastly, it is essential to consider the generalizability of our findings. The included studies may pertain to specific populations, interventions, or settings, thus potentially limiting the applicability of our results to other populations or clinical contexts.

## 5. Conclusions

Performing surgery for SOMs is intricate and challenging due to the tumor’s diffuse nature and its proximity to critical structures. The goals of surgical treatment for SOMs have undergone an evolution. Presently, the primary objective of surgical intervention is to safeguard visual function and ameliorate proptosis, rather than pursuing complete tumor resection. When visual compromise is evident, surgery has the potential to enhance and stabilize visual function.

To optimize patient outcomes, a multidisciplinary approach involving a skull base team is essential. This team comprises neurosurgeons, ophthalmologists, otorhinolaryngologists, maxillofacial surgeons, and radiologists. Their collaborative efforts yield several advantages, including early detection of optic nerve compromise, preoperative and postoperative evidence-based management, and improved surgical resection and clinical outcomes facilitated by the combined expertise of the team members.

## Figures and Tables

**Table 1:** Summary of studies included in the systematic literature review and meta-analysis. (CN = cranial nerve; GTR = gross total resection; NA = not available; RT = radiotherapy).

	Study					Baseline Data				Presentation				Treatment		Mean Follow-Up Time (Months)
No.	Author, Journal, Year	Title	Country	Prospective/Retrospective	Study Period	Sample Size	Mean Age at Intervention (range)	F (%)	WHO Grade (No.)	Visual Acuity Decrease No. (%)	CN Deficits (III, IV, VI) No. (%)	Proptosis No. (%)	Others	No. (%) of GTR	No. (%) of Patients Receiving Post-op RT	
1	Bonnal [8], J Neurosurg, 1980	Invading Meningiomas of the Sphenoid Ridge	Belgium	Retrospective	1958–1979	21	45 (23–65)	81%	NA	8 (38%)	3 (14%)	11 (52%)	Epilepsy, hemiparesis, aphasia, headache, intracranial hypertension, visual field deficit, Foster Kennedy syndrome, deafness, 5th and 6th nerve palsy	NA	NA	NA
2	Maroon [9], J Neurosurg, 1994	Recurrent Spheno-Orbital Meningioma	USA	Retrospective	1975–1992	15	46	73%	NA	6 (40%)	2 (13%)	13 (87%)	Blindness, visual field deficit, V1 hypesthesia	9 (60%)	10 (67%)	NA
3	Gaillard [10], Plastic and Reconstructive Surgery, 1997	Strategy of Craniofacial Reconstruction After Resection of Spheno-Orbital “en Plaque” Meningiomas	France	Retrospective	1981–1993	20	NA	NA	NA	NA	2 (10%)	NA	NA	NA	NA	84
4	De Jesus [11], Surg Neurol, 2001	Surgical Management of Meningioma en Plaque of the Sphenoid Ridge	Puerto Rico	Retrospective	1990–1997	6	51 (39–64)	100%	NA	3 (50%)	NA	5 (83%)	Seizure	5 (83%)	NA	48
5	Leake [12], Arch Facial Plast Surg, 2005	Reconstruction after Resection of Sphenoid Wing Meningiomas	USA	Retrospective	1995–2004	22	53 (31–73)	77%	NA	5	NA	15 (68%)	Visual field deficit, trigeminal hypoesthesia, seizure, dysphagia	11 (50%)	4 (18%)	15
6	Roser [13], Surg Neurol, 2005	Sphenoid Wing Meningiomas with Osseous Involvement	Germany	Retrospective	NA	82	53 (21–78)	77%	NA	18 (22%)	2 (2%)	31 (38%)	Headache, aphasia, trigeminal neuralgia, seizure	31 (38%)	NA	66
7	Shrivastava [14], J. Neurosurg, 2005	Spheno-Orbital Meningiomas: Surgical Limitations and Lessons Learned in Their Long-Term Management	USA	Retrospective	1991–2003	25	51 (22–76)	88%	NA	20 (80%)	5 (20%)	22 (88%)	Trigeminal hypoesthesia, scotoma	18 (70%)	2 (8%)	60
8	Sandalcioglu [15], Journal of Cranio- Maxillofacial Surgery, 2005	Spheno-orbital Meningiomas: Interdisciplinary Surgical Approach, Resectability and Long-Term Results	Germany	Retrospective	1998–2002	16	53 (3–76)	94%	I	7 (44%)	1 (6%)	14 (88%)	Diplopia	16 (100%)	2 (13%)	68
9	Schick [16], J Neurosurg, 2006	Management of Meningiomas en Plaque of the Sphenoid Wing	Germany	Retrospective	1991–2002	67	58 (32–79)	79%	I (64), II (3)	28 (42%)	11 (16%)	33 (49%)	V palsy, visual field deficit	40 (60%)	5 (7%)	46
10	Ringel [17], Operative Neurosurg, 2006	Microsurgical Technique and Results of a Series of 63 Spheno-orbital Meningiomas	Germany	Retrospective	1983–2003	63	51 (21–77)	79%	NA	28 (44%)	16 (25%)	50 (79%)	Visual field deficit, seizure, diplopia	45 (71%)	NA	54
11	Bikmaz [18], J Neurosurg, 2007	Management of Bone-Invasive, Hyperostotic Sphenoid Wing Meningiomas	USA	Retrospective	1994–2004	17	52 (36–70)	88%	NA	10 (59%)	3 (18%)	12 (71%)	Eye swelling, headache, incidental, diplopia	14 (82%)	NA	36
12	Yong [19], Chin Med J (Engl), 2009	Sphenoid Wing Meningioma en Plaque: Report of 37 Cases	China	Retrospective	1998–2009	37	46 (16–67)	59%	I (33), II (2), III (2)	26 (70%)	NA	37 (100%)	Headache, seizure	9 (24%)	10 (27%)	36
13	Scarone [20], J Neurosurg, 2009	Long-Term Results with Exophthalmos in a Surgical Series of 30 Spheno-Orbital Meningiomas	France	Retrospective	1994–2005	30	51 (35–74)	100%	NA	6 (20%)	NA	28 (93%)	Headache, temporal swelling, visual field deficit	27 (90%)	1 (3%)	NA
14	Heufelder [21], Ophthalmic Plastic and Reconstructive Surgery, 2009	Reconstructive and Ophthalmologic Outcomes Following Resection of Spheno-Orbital Meningiomas	Germany	Retrospective	1997–2006	21	61 (47–81)	95%	I (19), II (2)	NA	NA	18 (86%)	Visual field deficit, epiphora	NA	5 (24%)	66
15	Mirone [22], Neurosurgery, 2009	En Plaque Sphenoid Wing Meningiomas: Recurrence Factors and Surgical Strategies in a Series of 71 Patients	France	Retrospective	1986–2006	71	53 (12–79)	87%	I	41 (58%)	15 (21%)	61 (86%)	Diplopia, headache, trigeminal pain, visual field deficit, chemosis, seizure	59 (83%)	1 (1%)	77
16	Cannon [5], Orbit, 2009	The Surgical Management and Outcomes for Spheno-Orbital Meningiomas: A 7-Year Review of Multi-Disciplinary Practice	UK	Retrospective	2000–2007	12	51 (34–64)	92%	I (11), II (1)	5 (42%)	1 (8%)	12 (100%)	Diplopia	NA	3 (25%)	31
17	Civit [23], Neuro- chirurgie, 2010	Spheno-Orbital Meningiomas	France	Retrospective	NA	41	NA	NA	NA	23 (56%)	4 (9%)	39 (95%)	V deficit, visual field deficit	NA	NA	NA
18	Honig [24], Neurological research, 2010	Spheno-Orbital Meningiomas: Outcome After Microsurgical Treatment: A Clinical Review of 30 Cases	Germany	Retrospective	2001–2006	30	54 (25–74)	73%	I (26), II (3), III (1)	22 (73%)	6 (20%)	16 (53%)	Diplopia, headache, trigeminal pain, visual field deficit, chemosis, seizure	10 (33%)	8 (27%)	34
19	Oya [25], J Neurosurg, 2011	Spheno-Orbital Meningioma: Surgical Technique and Outcome	USA	Retrospective	1994–2009	39	49 (33–68)	87%	NA	21 (54%)	3 (8%)	39 (100%)	Diplopia, headache, trigeminal pain, visual field deficit	15 (38%)	4 (10%)	41
20	Luetjens [26], Clin Neurol Neurosurg, 2011	Bilateral Spheno-Orbital Hyperostotic Meningiomas with Proptosis and Visual Impairment: A Therapeutic Challenge. Report of Three Patients and Review of the Literature	Germany	Retrospective	NA	3	62 (49–70)	100%	I	3 (100%)	NA	3 (100%)	Vertigo, diplopia	2 (66%)	1 (33%)	28
21	Mariniello [27], Acta Neurochir (Wein), 2013	Surgical Unroofing of the Optic Canal and Visual Outcome in Basal Meningiomas	Italy	Retrospective	1986–2006	60	NA	NA	NA	60 (100%)	NA	NA	Visual field deficit, diplopia	NA	NA	60
22	Boari [28], British Journal of Neurosurgery, 2013	Management of Spheno-Orbital en Plaque Meningiomas: Clinical Outcome in a Consecutive Series of 40 Patients	Italy	Retrospective	2000–2010	40	53 (NA)	88%	NA	35 (88%)	2 (5%)	18 (45%)	Visual field deficit, diplopia	22 (56%)	18 (44%)	73
23	Saeed [29], Br J Ophthalmol, 2011	Surgical Treatment of Spheno-Orbital Meningiomas	Netherlands	Retrospective	1980–2006	66	46 (26–68)	92%	NA	51 (77%)	NA	66 (100%)	Diplopia, headache	39 (59%)	15 (23%)	102
24	Simas [30], Surg Neurol Int, 2013	Sphenoid Wing en Plaque Meningiomas: Surgical Results and Recurrence Rates	Portugal	Retrospective	1998–2008	18	52 (27–75)	83%	I (18)	5 (28%)	1 (6%)	16 (89%)	Temporal region swelling, orbital pain, diplopia, V1, V2 hypesthesia	7 (39%)	6 (33%)	55
25	Attia [31], World Neurosurg, 2013	Combined Cranio-Nasal Surgery for Spheno-Orbital Meningiomas Invading the Paranasal Sinuses, Pterygopalatine, and Infra-Temporal Fossa	USA	Retrospective	2009–2011	3	60 (44–82)	66%	I (2), II (1)	2 (67%)	1 (33%)	2 (67%)	V palsy	1 (33%)	1 (33%)	10
26	Marcus [32], Acta Neurochir (Wien), 2013	Image-Guided Resection of Spheno-Orbital Skull-Base Meningiomas with Predominant Intra- Osseous Component	UK	Retrospective	2004–2012	19	44 (25–64)	89%	I (17), II (2)	11 (58%)	6 (32%)	12 (63%)	Temporal swelling, headache, V paresthesia, focal sensory seizures	11 (58%)	2 (11%)	60
27	Mariniello [33], Clin Neurol Neurosurg, 2013	Management of the Optic Canal Invasion and Visual Outcome in Spheno-Orbital Meningiomas	Italy	Retrospective	1986–2006	60	NA	NA	NA	36 (60%)	19 (34%)	59 (98%)	Optic disc pallor, optic disc edema	40 (67%)	5 (8%)	NA
28	Forster [34], Neurol Res, 2014	Spheno-Orbital Meningiomas: Surgical Management and Outcome	Germany	Retrospective	2003–2013	18	50 (35–69)	100%	I (17), II (1)	7 (39%)	NA	15 (83%)	Diplopia, dizziness	13 (72%)	NA	44
29	Solmaz [35], Turk Neurosurg, 2014	Surgical Strategies for the Removal of Spheno-Orbital Meningiomas	Turkey	Retrospective	2006–2013	13	34 (26–58)	23%	I (13)	8 (62%)	NA	10 (77%)	Facial pain, orbital pain, epilepsy	4 (31%)	0	26
30	Talacchi [36], Neurosurg Rev, 2014	Surgical Management of Ocular Symptoms in Spheno-Orbital Meningiomas. Is Orbital Reconstruction Really Necessary?	Italy	Retrospective	1992–2012	47	57 (21–77)	56%	NA	24 (51%)	18 (32%)	46 (98%)	Periorbital and temporal swelling	24 (51%)	NA	52
31	Berhoum [37], Neurosurg Focus, 2014	Endoscopic Endonasal Optic Nerve and Orbital Apex Decompression for Nontraumatic Optic Neuro-pathy: Surgical Nuances and Review of the Literature	France	Retrospective	2012–2014	4	58 (49–67)	75%	NA	4 (100%)	NA	NA	Visual field deficit	NA	NA	6
32	Amirjamshidi [38], Surg Neurol Int, 2015	Lateral Orbito tomy Approach for Removing Hyper -ostosing en Plaque Sphenoid Wing Meningiomas. Description of Surgical Strategy and Analysis of Findings in a Series of 88 Patients with Long-Term Follow-up	Iran	Retrospective	1979–2013	88	46 (12–70)	74%	NA	65 (74%)	NA	88 (100%)	Visual field deficit, diplopia	NA	31 (35%)	135
33	Leroy [39], Acta Neurochir (Wein), 2016	Internal and External Spheno-Orbital Meningioma Varieties: Different Outcomes and Prog-noses	France	Retrospective	1995–2012	70	52 (21–80)	90%	I (60), II (5), III (5)	27 (39%)	NA	56 (80%)	Soft tissue tumefaction, headache, retrobulbar pain, whimpering, seizure, dizziness, diplopia	15 (11%)	18 (30%)	57
34	Bowers [40], J Neurosurg, 2016	Outcomes After Surgical Treatment of Meningioma-Associated Prop - tosis	USA	Retrospective	2002–2015	33	52 (12–76)	73%	NA	17 (52%)	NA	22 (22%)	Visual field deficit, diplopia, proptosis	31 (94%)	2 (6%)	54
35	Peron [41], Acta Neurochir Suppl, 2017	Spheno-Orbital Meningiomas: When the Endoscopic Approach is Better	Italy	Retrospective	2013–2014	30	46 (8–82)	73%	NA	1 (3%)	8 (27%)	21 (70%)	Visual field deficit, diplopia, V1 and V2 hypoesthesia	24 (80%)	NA	NA
36	Terrier [42], World Neurosurgery, 2017	Spheno-Orbital Meningiomas Surgery: Multicenter Management Study for Complex Extensive Tumors	France	Retrospective	1996–2016	130	51 (28–74)	92%	I	49 (38%)	13 (10%)	123 (95%)	Retro-orbital pain, diplopia, headache	97 (75%)	2 (2%)	77
37	Freeman [4], World Neurosurgery, 2017	Spheno-Orbital Meningiomas: A 16-Year Surgical Experience	USA	Retrospective	2000–2016	25	51 (39–71)	92%	I (21), II (5)	19 (76%)	NA	22 (88%)	Diplopia, headache, seizure	NA	11 (25%)	45
38	Gonen [43], Neurosurg Rev, 2017	Spheno-Orbital Meningioma: Surgical Series and Design of an Intra- Operative Management Algorithm	Israel	Retrospective	2005–2014	27	53 (27–78)	89%	NA	10 (37%)	4 (15%)	25 (92%)	Visual field deficit, diplopia, proptosis, seizure	14 (52%)	1 (3%)	41
39	Almeida [44], J Neurosurg, 2018	Trans-Orbital Endoscopic Eyelid Approach for Resection of Spheno-Orbital Meningiomas with Predominant Hyper-ostosis: Report of 2 Cases	USA	Retrospective	NA	2	59 (53–65)	100%	I (2)	2 (100%)	NA	2 (100%)	Visual field deficit	0 (0%)	2 (100%)	2
40	Belinsky [45], Ophthalmic Plast Reconstr Surg, 2018	Spheno-Orbital Meningiomas: An Analysis Based on World Health Organization Classification and Ki-67 Proliferative Index	USA	Retrospective	2000–2016	46	56 (27–85)	58%	I (30), II (4), III (4)	15 (33%)	4 (9%)	15 (33%)	seizure, altered mental status, double vision, epiphora, headache, V1 hypoesthesia	NA	25 (66%)	63
41	Dallan [46], Oper Neurosurg (Hagerstown), 2018	Endoscopic Trans- Orbital Superior Eyelid Approach for the Management of Selected Spheno-Orbital Meningiomas: Preliminary Experience	Italy	Retrospective	2012–2015	14	51 (35–73)	86%	I (14)	6 (43%)	2 (14%)	14 (100%)	Diplopia, pain, epiphora	3 (21%)	0 (0%)	25
42	Kong [47], J Neurosurg, 2018	Clinical and Ophthalmological Outcome of Endoscopic Trans-Orbital Surgery for Cranio- Orbital Tumors	Korea	Retrospective	2016–2017	12	56 (38–73)	92%	NA	7 (58%)	7 (39%)	14 (78%)	NA	4 (33%)	NA	5
43	Pace [48], Ophthalmic Plast Reconstr Surg, 2019	Orbital Reconstruction via Deformable Titanium Mesh Following Spheno-Orbital Meningioma Resection: Ophthalmic Presentation and Outcomes	USA	Retrospective	1996–2017	20	56 (19–89)	80%	NA	9 (45%)	3 (15%)	20 (100%)	Diplopia, visual field deficit	15 (75%)	4 (20%)	47
44	Nagahama [3], World Neurosurg, 2019	Spheno-Orbital Meningioma: Surgical Outcomes and Management of Recurrence	Japan	Retrospective	1996–2017	12	49 (20–71)	58%	I (15), II (2)	3 (25%)	NA	11 (92%)	Trigeminal hypoesthesia	3 (23%)	1 (8%)	74
45	De Rosa [49], Acta Neurochir (Wien), 2019	Endoscopic Endo- and Extra- Orbital Corridors for Spheno-Orbital Region: Anatomic Study with Illustrative Case	Italy	Retrospective	NA	1	37	100%	NA	0	0	1 (100%)	Lateral nystagmus, hypesthesia V1	NA	NA	6
46	Shapey [1], Acta Neurochir (Wien), 2019	A Single Centre’s Experience of Managing Spheno-Orbital Meningiomas: Lessons for Recurrent Tumour Surgery	London	Retrospective	2005–2016	31	49 (44–58)	65%	I (23), II (11)	13 (38%)	6 (18%)	13 (38%)	Diplopia, seizures, headaches, trigeminal pain, confusion/ somnolence	29 (85%)	4 (11,8%)	52
47	Young [6], Orbit, 2019	Combined NeuroSurgical and Orbital Intervention for Spheno-Orbital Meningiomas—the Manchester Experience	UK	Retrospective	2000–2017	24	50 (NA)	92%	I (23), II (1)	17 (71%)	3 (13%)	21 (88%)	Diplopia, headache, visual field deficit	0 (0%)	7 (29%)	82
48	Menon [50], J Neurosci Rural Pract, 2020	Spheno-Orbital Meningiomas: Optimizing Visual Outcome	India	Retrospective	10 years	17	51 (17–72)	76%	I (14) e II (3)	14 (82%)	NA	14 (82%)	Headache, facial paresthesia	2 (12%)	15 (88%)	56
49	Goncalves [51], J Neurol Surg B Skull Base, 2020	Trans-Orbital Endoscopic Surgery for Sphenoid Wing Meningioma: Long-Term Outcomes and Surgical Technique	South Africa	Retrospective	2015–2019	21	48,8 (34–79)	95%	I (20), II (1)	21 (100%)	1 (5%)	20 (95%)	Headache, facial pain, diplopia, blocked nose, epiphora	NA	1 (5%)	12
50	Park [52], World Neurosurg, 2020	Comparative Analysis of Endoscopic Trans-Orbital Approach and Extended Mini-Pterional Approach for Sphenoid Wing Meningiomas with Osseous Involvement: Preliminary Surgical Results	Republic of Korea	Retrospective	2015–2019	24	54 (24–73)	67%	NA	NA	NA	NA	Headache, cognitive decline, diplopia	21 (88%)	NA	20
51	Parish [53], J Neurol Surg Rep, 2020	Proptosis, Orbital Pain, and Long-Standing Monocular Vision Loss Resolved by Surgical Resection of Intra- Osseous Spheno-Orbital Meningioma: A Case Report and Literature Review	USA	Retrospective	2013	1	43	100%	NA	1 (100%)	NA	1 (100%)	Headache, periorbital pain	NA	NA	12
52	Samadian, World Neurosurg, 2020	Surgical Outcomes of Spheno-Orbital en Plaque Meningioma: A 10-Year Experience in 57 Consecutive Cases	Iran	Retrospective	2007–2017	57	48 (22–76)	93%	NA	16 (28%)	NA	47 (83%)	Visual field deficit, diplopia	48 (84%)	6 (11%)	46
53	Zamanipoor Najafabadi [54], Acta Neurochirurgica (Wein), 2021	Visual Outcomes Endorse Surgery of Patients with Spheno-Orbital Meningioma with Minimal Visual Impairment or Hyperostosis	Netherlands	Retrospective	2015–2019	19	47 (45–50)	95%	I	10 (53%)	NA	16 (84%)	Diplopia, headache, visual field deficit	14 (76%)	3 (16%)	46
54	In Woo [55], Graefes Arch Clin Exp Ophthalmol, 2021	Orbital Decompressive effect of Endoscopic Transorbital Surgery for Spheno-Orbital Meningioma	South Korea	Retrospective	2016–2019	18	54 (38–72)	89%	I (16), II (1)	10 (56%)	4 (22%)	17 (94%)	Visual field deficit	3 (17%)	12 (67%)	20
55	Masalha [56], Front Oncol, 2021	Progression-Free Survival, Prognostic Factors, and Surgical Outcome of Spheno-Orbital Meningioma	Germany	Retrospective	2000–2020	65	55	77%	I (52), II (13)	NA	NA	NA	NA	26 (40%)	15 (23%)	120
56	Dalle Ore [57], J Neurosurg, 2021	Hyperostosing Sphenoid Wing Meningiomas: Surgical Outcomes and Strategy for Bone Resection and Multidisciplinary Orbital Reconstruction	USA	Retrospective	NA	54	52 (30–79)	83%	I (45) e II (9)	28 (52%)	NA	40 (74%)	Visual field deficit, proptosis, diplopia	11 (20%)	18 (33%)	31
57	Gomes dos Santos [58], Surg Neurol Int, 2022	Spheno-Orbital Meningiomas: Is Orbit Reconstruction Mandatory? Long-Term Outcomes and Exophthalmos Improvement	Brazil	Retrospective	2008–2018	40	50 (NA)	88%	I (39) e II (1)	26 (65%)	8 (20%)	36 (90%)	Visual field deficit, headaches	26 (65%)	10 (25%)	39
58	Locatelli [59], J Neurol Surg B Skull Base, 2022	The Role of the Trans-Orbital Superior Eyelid Approach in the Management of Selected Spheno-Orbital Meningiomas: In-Depth Analysis of Indications, Technique, and Outcomes from the Study of a Cohort of 35 Patients	Italy	Retrospective	2011–2021	35	57 (38–80)	77%	I (31), II (4)	11 (32%)	7 (20%)	22 (63%)	Visual field deficit, proptosis, diplopia, seizure	16 (46%)	NA	32
59	Wierzbowska [2], J Clin Med, 2023	Spheno-Orbital Meningioma and Vision Impairment—Case Report and Review of the Literature	Poland	Retrospective	NA	1	46	100%	I	Yes	No	Yes	NA	1 (100%)	NA	78

**Table 2:** Overall efficacy and safety outcomes.

	Overall % (95%CI)
GTR	57.3% (47.5–67.1)
Recurrence	20.7% (16.6–24.8)
PFS 5-y	75.5% (70.0–81.1)
PFS 10-y	49.1% (41.3–56.8)
Vision acuity improvement	57.5% (51.7–63.3)
Proptosis improvement	79.3% (73.7–84.8)
CN focal deficits	20.6% (14.9–26.3)
CSF leak	3.9% (2.3–5.5)
Other	13.9% (10.1–17.7)

**Table 3:** Subgroups efficacy and safety outcomes.

	MTA	ETOA	ETOA + EEA	*p* Value
	% (95%CI)	% (95%CI)	% (95%CI)	
GTR	59.8 (49.5–70.2)	41.3 (11.6–70.9)	23.5 (0–52.5)	0.001
Recurrence	24.4 (19.4–29.4)	4.4 (0–11.2)	NA	0.014
Vision acuity improvement	57.3 (51–63.5)	69.2 (41.5–96.9)	51.3 (16.7–85.9)	0.902
Proptosis improvement	60 (47.4–72.6)	79.4 (57.3–100)	69.8 (37.0–100)	0.002
CN focal deficits	21 (14.5–27.6)	7.3 (0–18.1)	20.3 (0–46.7)	0.411
CSF leak	4.9 (2.8–6.9)	5 (0–11.6)	20.3 (0–46.7)	0.551
Other	13.6 (9.5–17.7)	15.4 (1.6–29.2)	NA	0.866
	**WHO Grade** **I**	**WHO Grades** **I + II**	**WHO Grades** **I + II + III**	***p* Value**
	**% (95%CI)**	**% (95%CI)**	**% (95%CI)**	
GTR	43.1 (20.4–65.9)	46.5 (26.8–66.1)	57.3 (47.5–67.1)	0.001
Recurrence	17.7 (1.6–33.9)	24.8 (14.9–34.7)	20.7 (16.6–24.8)	0.185
Vision acuity improvement	69.0 (47.6–90.4)	54.7 (41.1–68.3)	57.5 (51.7–63.3)	0.779
Proptosis improvement	77.3 (60.9–93.7)	74.0 (61.3–86.6)	79.3 (73.7–84.8)	0.013
CN focal deficits	12.4 (6.9–17.9)	15.4 (6.7–24.2)	20.6 (14.9–26.3)	0.224
CSF leak	5 (0–11.8)	5.2 (1.2–9.2)	3.9 (2.3–5.5)	0.983
Other	22.1 (5.1–39.2)	11.7 (6.5–16.8)	13.9 (10.1–17.7)	0.001
	**Anatomical Class I**	**Anatomical Class I + II + III + IV**		***p* Value**
	**% (95%CI)**	**% (95%CI)**		
GTR	78.6 (60.1–97.1)	57.3 (47.5–67.1)		0.001
Recurrence	15.1 (6.5–23.7)	20.7 (16.6–24.8)		0.001
Vision acuity improvement	71.5 (63.7–79.4)	57.5 (51.7–63.3)		0.003
Proptosis improvement	60.1 (38–82.2)	79.3 (73.7–84.8)		0.001

## Data Availability

Data available in a publicly accessible repository.

## Abbreviations

Cerebro-spinal fluid (CSF), confidential intervals (CIs), cranial nerve (CN), endoscopic endonasal approaches (EEAs), endoscopic transorbital approaches (ETOAs), external beam radiotherapy (EBRT), gross total resection (GTR), intensity-modulated radiotherapy (IMRT), microsurgical transcranial (MTAs), spheno-orbital meningiomas (SOMs), Preferred Reporting Items for Systematic Reviews and Meta-Analysis (PRISMA), progression-free survival (PFS), stereotactic radiosurgery (SRS).
